# Functional Metagenomics of *Escherichia coli* O157:H7 Interactions with Spinach Indigenous Microorganisms during Biofilm Formation

**DOI:** 10.1371/journal.pone.0044186

**Published:** 2012-09-05

**Authors:** Michelle Q. Carter, Kai Xue, Maria T. Brandl, Feifei Liu, Liyou Wu, Jacqueline W. Louie, Robert E. Mandrell, Jizhong Zhou

**Affiliations:** 1 Produce Safety and Microbiology Research Unit, Western Regional Research Center, Agricultural Research Service, United States Department of Agriculture, Albany, California, United States of America; 2 Institute for Environmental Genomics, Department of Botany and Microbiology, University of Oklahoma, Norman, Oklahoma, United States of America; 3 Department of Environmental Science and Engineering, Tsinghua University, Beijing, China; 4 Earth Sciences Division, Lawrence Berkeley National Laboratory, Berkeley, California, United States of America; U. S. Salinity Lab, United States of America

## Abstract

The increase in foodborne outbreaks worldwide attributed to fresh fruit and vegetables suggests that produce may serve as an ecological niche for enteric pathogens. Here we examined the interaction of *E. coli* O157:H7 (EcO157) with spinach leaf indigenous microorganisms during co-colonization and establishment of a mixed biofilm on a stainless steel surface. Stainless steel surface was selected to mimic the surface of produce-processing equipment, where retention of foodborne pathogens such as EcO157 could serve as a potential source for transmission. We observed a positive effect of spinach-associated microbes on the initial attachment of EcO157, but an antagonistic effect on the EcO157 population at the later stage of biofilm formation. Metagenomic analyses of the biofilm community with the GeoChip revealed an extremely diverse community (gene richness, 23409; Shannon-Weiner index H, 9.55). Presence of EcO157 in the mixed biofilm resulted in a significant decrease in the community α-diversity (t test, *P*<0.05), indicating a putative competition between the pathogen and indigenous spinach microbes. The decrease in the β-diversity of the EcO157-inoculated biofilm at 48 h (ANOVA, *P*<0.05) suggested a convergent shift in functional composition in response to EcO157 invasion. The success of EcO157 in the mixed biofilm is likely associated with its metabolic potential in utilizing spinach nutrients: the generation time of EcO157 in spinach lysates at 28°C is ∼ 38 min, which is comparable to that in rich broth. The significant decrease in the abundance of many genes involved in carbon, nitrogen, and phosphorus cycling in the EcO157-inoculated biofilms (t test, *P*<0.05) further support our conclusion that competition for essential macronutrients is likely the primary interaction between the EcO157 and indigenous spinach-biofilm species.

## Introduction

*E. coli* O157:H7 (EcO157) is a common Enterohemorrhagic *Escherichia coli* (EHEC) serotype that contributes significantly to human infections and outbreaks world-wide. EHEC infections linked to fresh produce have accounted for at least 40 reported *E. coli* outbreaks in the US since 1982 [1]. For example, bagged spinach was the source of the 2006 US outbreak of EcO157 infections; shredded Romaine lettuce was linked to the 2010 US outbreak of *E. coli* O145 infections; and fenugreek sprouts was likely the vehicle of the 2011 large outbreak of *E. coli* O104 infections in Germany (http://www.cdc.gov/ecoli/). These produce-associated outbreaks suggest that plants may serve as an important ecological niche for enteric pathogens.

Fresh vegetables can become contaminated at multiple points in the food processing chain, thus, knowledge of survival and persistence of enteric pathogens on edible crops and during produce production provides information for agricultural practices. Leaves are inhabited by diverse highly-adapted microbes and are colonized frequently by transient residents, including human pathogens [2]. Complex interactions may exist between human pathogens and the indigenous microflora associated with fresh produce [3], [4], [5]. Such interactions may determine the fate of enteric pathogens on produce in pre- and post-harvest environments and are likely affected by the microenvironments where they occur. Leafy vegetables are rich in nutrients [6], some of which may serve as substrates for growth of enteric pathogens, thus, EcO157 populations may amplify rapidly in response to nutrients leaked from damaged leaves. Indeed, the rapid growth of EcO157 in lettuce leaf lysates [7] and on cut lettuce leaves [8] has been reported. Leakage of nutrients from damaged plant cells is inherent to the harvest and processing of leafy vegetables. Considering the diversity and complexity of the plant-associated microbial community [2], [9], [10], a better understanding of dynamics at the whole community level is needed to identify factors involved in the survival and persistence of enteric pathogens on produce and in produce production environments.

High-throughput metagenomic technology has provided powerful tools for examination of microbial interactions at the community level in greater depth. The GeoChip is a gene array designed to study the functional structure and metabolic diversity of a microbial community [11], [12] and has been used widely to assess the functional communities of various ecosystems [13], [14], [15], [16], [17]. The newest version, GeoChip 4.0, contains more than 82,000 probes representing genes from over 400 functional categories [13], [15]. Here, we examined the impact of spinach-associated microorgansims on the growth of, and biofilm formation by, EcO157. We characterized the interactions between EcO157 and the spinach indigenous microbes during co-colonization of a stainless steel coupon (SSC). SSCs are used commonly for studies of biofilms associated with food processing; thus, it was selected to mimic the surface of produce-processing equipment, where retention of foodborne pathogens such as EcO157 could serve as a potential source for transmission. Our results indicate a positive- and negative effect of spinach-associated microorganisms on the EcO157 biofilm population in the early- and late stage of a mixed biofilm, respectively. A significant decrease in the abundance of many genes involved in nutrient cycling in the mixed biofilm suggested that competition was the dominant interaction between EcO157 and spinach-associated microbes in the biofilm.

## Materials and Methods

### Bacterial Strains and Growth Conditions

The EcO157 strain (MB526) used in this study is a rifampin-resistant spontaneous mutant of RM6067, a bagged-spinach isolate linked to the 2006 US spinach-associated outbreak (provided by E. Hyttia-Trees, CDC). MB526 was maintained on Tryptic Soy Agar (TSA) supplemented with rifampin (100 µg ml^−1^).

### Preparation of Spinach Leaf Lysates

Fresh spinach leaf lysates were prepared on the day of the experiment from bagged ready-to-eat organic baby spinach as described previously [7]. Briefly, the spinach leaves were homogenized using an Omega 8003 juicer (Omega Products, Inc., Harrisburg, PA). The homogenized sample was centrifuged twice at 10,000 g for 10 min to pellet the plant debris. The supernatant, hereafter called spinach lysate, was used undiluted or diluted with sterile water. Sterile lysates were prepared by filtration through a 0.45 µm-pore-size filter first, then through a 0.2 µm-pore-size filter. Sterility of the lysates was verified by plating 100 µl of filtered lysates onto TSA and incubating the plates at 28°C for at least two days.

### Growth of EcO157 in Spinach Leaf Lysates

An overnight culture of EcO157 (grown in 5% TSB at 28°C) was collected by centrifugation (10,000 g, 3 min), washed once and re-suspended in saline solution (0.85% NaCl). Spinach lysates were inoculated at 5×10^3^ cells ml^−1^ and incubated at 28°C with aeration (150 rpm). The growth of EcO157 was monitored over a 48-h period by dilution plating onto Rif-TSA plates using a spiral plater (Autoplate®4000, Spiral Biotech, Norwood, MA). A control without EcO157 inoculation was used to monitor for natural rifampin-resistant bacteria in the lysates.

### Mixed Biofilm with EcO157

Precut 1.0× 0.5 inch SSCs (Speedy Metals LLC, New Berlin, WI) were cleaned and sterilized as described previously [18]. A sterile coupon was placed in a culture tube containing two ml of spinach lysates inoculated with 5×10^3^ EcO157 cells ml^−1^, and incubated at 28°C with gentle shaking (80 rpm). At each sampling time, the coupon was removed and rinsed twice with saline solution. The total biomass on the coupon was removed using a cotton swab and re-suspended in one ml of saline solution. The cell suspension was mixed by vortexing and sonication before dilution plating onto Rif-TSA plates.

### GeoChip-based Metagenomic Study

The spinach control biofilms were produced on SSCs in non-sterile 100% spinach lysates without inoculation of EcO157, thus designated as 24-C and 48-C for samples collected at 24- and 48 h, respectively. The mixed biofilms with EcO157 were set up under the same conditions as control biofilms, except that spinach lysates were inoculated with 5×10^3^ EcO157 cells ml^−1^, and were designated as 24-S and 48-S, correspondingly. Three biological replicates were collected for each biofilm community examined. The biofilm cells were centrifuged (18,000 g, 5 min) and the pellets were stored at −80°C until DNA extraction with a Wizard® Genomic DNA Purification kit (Promega). 2 µg of genomic DNA from each replicate sample was labeled with fluorescent dye Cy-3 using random primers [19]. The labeled gDNA was dried and rehydrated with 2.7 µl of sample tracking control, followed by incubation at 50°C for 5 min. This DNA solution was then mixed with 7.3 µl of hybridization buffer containing the universal standard DNA labeled with Cy-5 dye, denatured at 95°C for 5 min, and maintained at 42°C until loaded onto GeoChip arrays (NimbleGen, Madison, WI). The hybridization was performed on a Hybridization Station (MAUI, Roche, CA) at 42°C for 16 h with agitation. After washings, the arrays were scanned using a MS 200 Microarray Scanner (NimbleGen) at laser power of 100% PMT (photomultiplier tube).

### Data Pre-processing and Analyses

The signal intensity of each spot on the GeoChip was first normalized across samples by the mean of Cy-5 labeled universal standard signal intensities and then by the mean of Cy-3 labeled signal intensities of all hybridized spots within each smaple. The spots were scored as positive if the signal-to-noise ratio (SNR) was >2.0 and the coefficient of variation (CV) of the background was <0.8. The normalized data were then subjected to statistical analyses using the Vegan package in R 2.9.1 (The R Foundation for Statistical Computing, Vienna, Austria) and the pipeline developed at University of Oklahoma (http://ieg.ou.edu). Detrended correspondence analysis (DCA) was applied to assess the overall functional compositions of biofilm communities [20]. The impact of EcO157 was assessed by comparing the community α-diversity between the control- and the EcO157-inoculated biofilms. The dissimilarities among replicate samples in control or EcO157-inoculated biofilm communities were assessed based on Euclidean β-diversity index. Permutation analysis of variance (PANOVA) was performed to test whether the influence of EcO157 was significant for community α- or β diversity. The *post-hoc* least significant distance (LSD) test with Holm–Bonferroni adjustment was performed only if the impact of EcO157 was time-dependent based on the PANOVA test. GeoChip data are available at http://ieg.ou.edu/4download.

## Results

### Growth of EcO157 in Spinach Lysates

**Figure 1:**
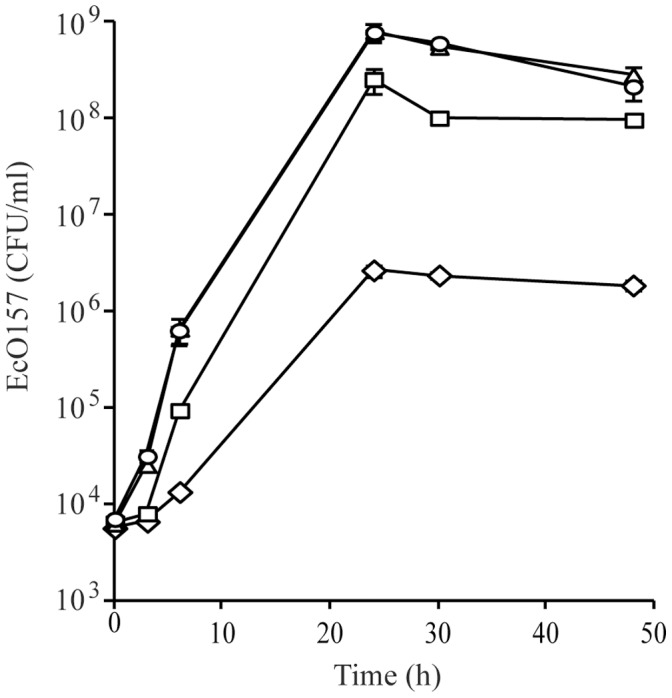
Growth of EcO157 in spinach lysates. The concentration of EcO157 was determined in filter-sterilized 100% spinach lysates (○); non-sterile 100% spinach lysates (Δ); non-sterile 5% spinach lysates (□); and non-sterile 0.1% spinach lysates (◊). Each datum point represents the mean bacterial concentration in three replicate cultures. Most bars representing standard deviation are too small to show.

To examine the impact of spinach-associated microbes on the growth of EcO157 in spinach lysates, the pathogen was inoculated into non-sterile or filter-sterilized freshly-made spinach lysates and its total population was monitored over 48 h (Figure 1). Rapid growth of EcO157 was observed in full strength sterile and non-sterile spinach lysates with generation times of 42- and 38 min, and maximum population sizes of 7.7×10^8^ and 7.9×10^8^ CFUs ml^−1^ at 24 h, respectively. Thus, the presence of spinach-associated microorganisms did not appear to have considerable impact on the growth of EcO157. In contrast, the growth of EcO157 in spinach lysates appeared to be largely affected by the concentration of the lysates, with generation times of 51- and 176 min, and population sizes of 2.5×10^8^ and 2.7×10^6^ CFU ml^−1^ at 24 h in 5.0- and 0.1% non-sterile lysates, respectively (Figure 1).

### Mixed Biofilm Formation on SSC

**Figure 2:**
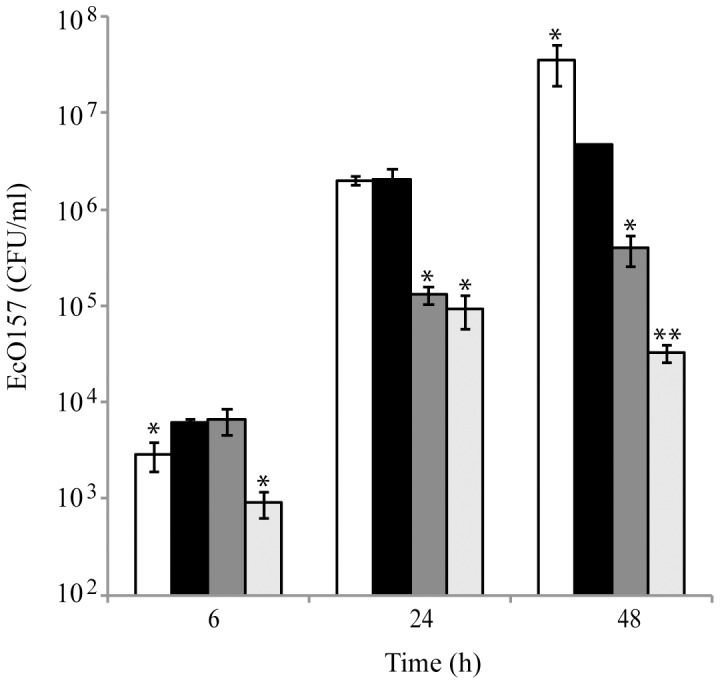
Biofilm formation of EcO157 on a stainless steel surface in spinach lysates. The EcO157 biofilm population size was measured in filter-sterilized 100% spinach lysates (white columns); non-sterile 100% spinach lysates (black columns); non-sterile 5% spinach lysates (dark grey columns); and non-sterile 0.1% spinach lysates (light grey columns). Data represent the mean EcO157-biofilm population size and standard deviation of the mean in three replicate lysates. At each time point, the mean population size of EcO157 in the biofilms was compared with that in non-sterile 100% spinach lysates by ANOVA (**P<0.01; *P<0.05).

The population size of EcO157 in the biofilm on a SSC was measured after 6, 24 and 48 h incubation in the four different spinach lysates described above. At 6 h, the EcO157 biofilm population size was significantly greater in the non-sterile than in the sterile lysates (Figure 2, ANOVA, *P* = 0.009). At 24 h, no significant difference was observed between the EcO157-biofilm population sizes in the above two growth conditions, however, at 48 h, the EcO157-biofilm population size in the non-sterile spinach lysates was much lower than that in the sterile-spinach lysates (Figure 2, ANOVA, *P* = 0.03). These data suggest a role for the spinach-associated microorganisms in EcO157-biofilm formation on SSCs. Similarly to the planktonic growth of EcO157 in spinach lysates, EcO157- biofilm population sizes varied significantly with the concentration of the lysate at various times after inoculation (Figure 2 and Table S1).

### The Functional Composition of the Spinach Biofilm as Detected by GeoChip

About 27% and 21% of the total probes deposited on the GeoChip were detected in the control spinach biofilm at 24 h (24-C) and 48 h (48-C), respectively. These included (i) genes conferring resistance to antibiotics or heavy metals; (ii) genes related to stress, virulence or bioremediation; (iii) genes involved in energy production, carbon, nitrogen, phosphorus, or sulphur cycling/utilization; and (iv) bacterial phage genes (Figure S1).

There was considerable functional gene richness for genes conferring stress tolerance or metal resistance, and genes involved in carbon- and nitrogen cycling (Table S2). For example, 44 genes detected in 24-C biofilms were associated with a role in cell survival under nutrient limitation or adverse physiological conditions such as oxidative and osmotic stress, heat shock, *etc*. (Figure S1, C), whereas 43 genes were related to heavy metal resistance (Figure S1, B). Furthermore, we detected 41- and 15 key genes involved in carbon- or nitrogen cycling, respectively (Figure S1, F and G). For carbon cycling, about 78% of detected DNA probes represented genes associated with carbon degradation. Other detected genes involved in nutrient transformation included genes encoding a phytase, a polyphosphate kinase, and a phosphatase for phosphorus cycling; an adenosine-5′-phosphosulfate reductase, a sulfite reductase, and a sulphur oxidase, for sulphur utilization (Figure S1, H and I). Additionally, three major groups of antibiotic resistance genes were detected, including those for multidrug transporters, a beta-lactamase, and a tetracycline resistance gene (Figure S1, A). Many of the virulence-related genes encoding adhesion/colonization factors or iron acquisition proteins were in high abundance (Figure S1, D). Overall, the signal intensity per functional category declined in 48-C biofilms. However, genes involved in nitrification or energy production, genes related to glucose limitation, and bacterial phage genes playing a role in host cell recognition and lysis, increased in the control biofilms over time (Table S2).

### The *gyrB*-based Phylogenetic Composition of Spinach Biofilm Communities

748 *gyrB* probes from diverse phylogenetic groups were detected in spinach biofilm communities. Based on the signal intensities of *gyrB*, *Proteobacteria* was the major bacterial phylum detected in 24-C and 48-C biofilms. α- and γ *Proteobacteria* were the dominant groups, followed by β-, δ-, and ε *Proteobacteria*. Other abundant bacterial phyla were *Actinobacteria* and *Firmicutes*, followed by *Bacteroidetes*, *Cyanobacteria*, and a large number of unclassified or uncultured bacteria. Significantly lower signal intensities for α-, β-, and γ *Proteobacteria*, *Actinobacteria*, *Planctomycetes*, and *Chlorobi* were observed in the 48-C compared with the 24-C biofilm community, whereas the phylum *Thermobaculum* showed enhanced hybridization signal intensities in the 48-C biofilm community (Figure S2 and Table S3).

### Impact of EcO157 on Spinach Biofilm Communities

**Figure 3:**
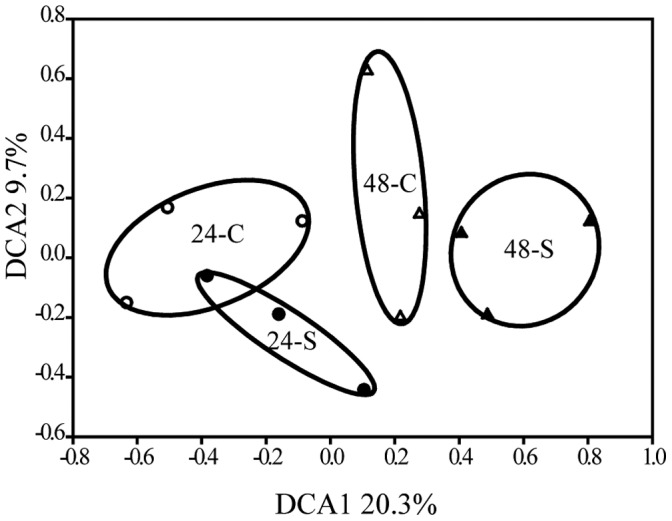
Detrended correspondence analysis (DCA) of control- and EcO157-inoculated spinach biofilm communities based on the all detected probes. 24-C, control biofilm community at 24 h (○); 48-C, control biofilm community at 48 h (Δ); 24-S, EcO157-inoculated biofilm community at 24 h (•); 48-S, EcO157-inoculated biofilm community at 48 h (▴).

The community-wide response to EcO157 during colonization and establishment of the mixed biofilm was examined by comparing the functional gene composition of control biofilms (24-C and 48-C) with those of mixed biofilms with EcO157 (24-S and 48-S). The DCA profile showed that the EcO157-inoculated biofilm began to diverge from the control biofilm along both axes at 24 h, despite the two communities (24-C and 24-S) overlapping considerably (Figure 3). However, at 48 h, the EcO157-inoculated biofilm (48-S) separated completely from the control biofilm (48-C) along the DCA1, indicating a substantial change in functional gene composition (Figure 3).

We then compared the community α-diversity between the control- and EcO157-inoculated biofilms. Significant decreases in gene richness (15.5%, t test *P* = 0.05) and diversity based on Shannon-Weiner index were observed in 24-S biofilms compared with 24-C biofilms, whereas, a significant decline only in the gene richness occurred in 48-S biofilms (18.3%, t test, *P* = 0.043) (Table 1). Based on Euclidean index analysis, there was no significant difference in the β-diversity of 24-S and 24-C biofilms. However, the β-diversity of 48-S biofilms decreased significantly compared with that of the 48-C (28.6%, ANOVA *P*<0.05) (Table 2). PANOVA revealed that the impact of EcO157 was significant on the β-diversity of the biofilm and that this effect was time-dependent (PANOVA, EcO157, *P* = 0.001; interaction, *P* = 0.01) (Table 2).

**Table 1:** The impact of EcO157 on biofilm community α-diversity.

α-diversity indices	Biofilm community^a^				*P* value (t test)^b^	
	24-C	24-S	48-C	48-S	24 h	48 h
Richness^c^	23409±1203	19778±1212	17662±313	14423±1394	0.050	0.043
Shannon-Weiner (H)	9.55±0.06	9.37±0.06	9.26±0.08	9.12±0.07	0.048	0.119

a mean ± standard error, n = 3;

b 24 h, t test between 24-C and 24-S; 48 h, t test between 48-C and 48-S;

c total number of probes detected by GeoChip4.0.

**Table 2:** The impact of EcO157 on biofilm community β-diversity.

β-diversity	Biofilm community^a^				*P* value (PANOVA)^b^		
	24-C	24-S	48-C	48-S	EcO157	Time	Interaction
Euclidean	146.0±2.6b	141.7±0.5b	183.2±8.7a	130.8±10.2b	0.001	0.108	0.010

a The β-diversity (mean ± standard error, n = 3) of each biofilm community was calculated based on Euclidean index. Different letters after the means indicate statistical difference at *P*<0.05 by least significant distance (LSD) test with Holm–Bonferroni adjustment when the interaction effect was significant by PANOVA.

b Permutational analysis of variance (PANOVA) of β-diversity to test for effect of EcO157, time, and their interaction on community dissimilarity.

Examination of individual genes on the GeoChip revealed that 85- and 49 genes declined significantly in hybridization signal intensities in the EcO157-inoculated biofilms at 24- and 48 h, respectively, compared with control biofilms (t test, *P*<0.05) (Table S4). Many of these genes encode functions involved in metabolic processes and nutrients cycling. Genes associated with utilization of carbon, nitrogen, and phosphorus were analyzed in detail.

### Carbon

**Figure 4:**
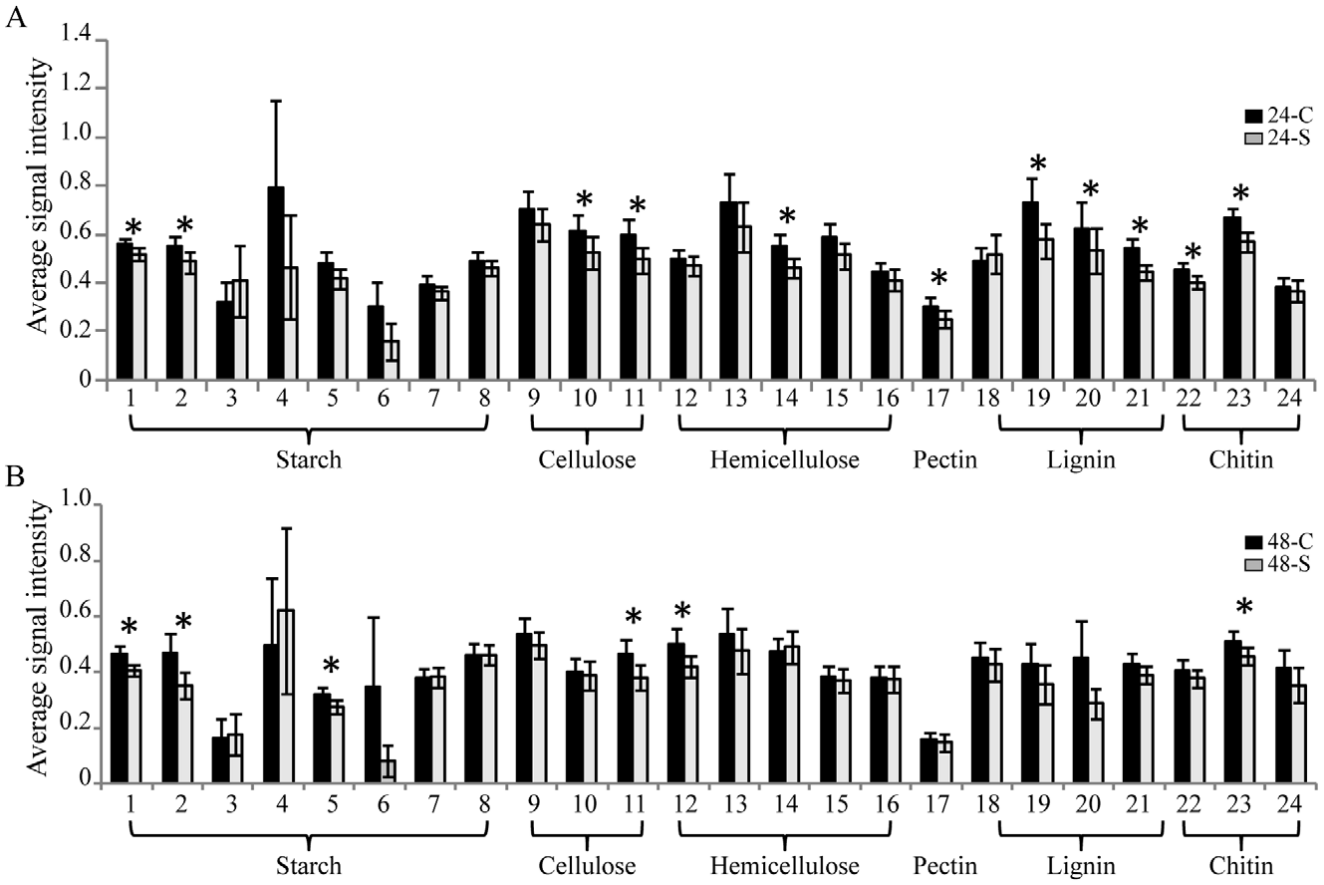
The impacts of EcO157 on genes involved in carbon degradation in spinach biofilm communities. Panels A and B are biofilms at 24 h and 48 h, respectively. The abundance of each gene is expressed as the average signal intensity of all gene probes detected for that gene in each biofilm community. Error bar represent the standard error of the mean for each gene. Genes with a significant decrease in the abundance between the control- and the EcO157-inoculated biofilm communities were marked with an asterisk (*) (t test, *P*<0.05). Starch degradation: 1, α-amylase (*amyA*); 2, glucoamylase; 3, amylopullulanase (*amyX*); 4, amylopullulanase (*apu*); 5, pullulanase (*pulA*); 6, isopullulanase; 7, neopullulanase II (*nplT*); 8, dextrin-hydrolase (*cda*). Cellulose degradation: 9, endoglucanase (*egl*); 10, exoglucanase; 11, cellobiase or β-glucosidase (*bgl*). Hemicelluloses degradation: 12, bacterial arabinofuranosidase (*ara*); 13, fungal arabinofuranosidase (*ara*); 14, mannanase; 15, xylose isomerase (*xylA*); 16, xylanase. Pectin degradation: 17, pectinase. Lignin degradation: 18, glyoxal oxidase (*glx*); 19, ligninase (*lip*); 20, manganese peroxidase (*mnp*); 21, phenol oxidase (*lcc*). Chitin degradation: 22, acetylglucosaminidase; 23, endochitinase; 24, exochitinase.

Strong signal intensities were detected in 24-C and 48-C biofilms for 24 genes encoding enzymes that degrade various complex carbon polymers, including starch, cellulose, hemicelluloses, pectin, lignin, and chitin (Figure 4). The abundance of 11 and 6 genes decreased significantly in EcO157-inoculated biofilms compared with in control biofilms at 24- and 48 h, respectively (Figure 4). Because each gene on the GeoChip is represented by DNA probes derived from diverse phylogenetic groups, we compared the signal intensities of each DNA probe in the EcO157-inoculated biofilms with that in the control biofilms to reveal the EcO157-induced responses from the carbon polymer-degrading members in the biofilm community.

#### Starch

Among the eight genes involved in breaking down starch to simple sugars, the abundances of the α-amylase and the glucoamylase genes decreased significantly in 24-S and 48-S biofilms, whereas the pullulanase gene decreased significantly only in 48-S biofilms compared with 48-C biofilms (Figure 4, starch). The majority of DNA probes targeting these three genes were from *Proteobacteria* (233 genes probes), *Actinobacteria* (156 gene probes), and *Firmicutes* (67 gene probes), however, the abundance of DNA probes from *Actinobacteria* decreased significantly compared with those in control biofilms only at 48 h (Table 3, Starch). No significant difference in signal intensity of *Proteobacteria* DNA probes between the control- and EcO157-inoculated biofilms was observed, regardless of their high abundance (Table S5).

**Table 3:** Phylogenetic origins of carbon, nitrogen, and phosphorus cycling genes affected significantly by the presence of EcO157 in the biofilm.

Functional group	Phylum	Normalized signal intensity^a^				
		24-C	24-S	48-C	48-S	
*Carbon degradation*						
Starch	*Actinobacteria*	104.1	92.7	89.0	66.0**	
Cellulose	*Actinobacteria*	34.9	27.1*	24.5	15.6**	
	*Firmicutes*	6.7	5.2*	6.9	7.0	
	Uncultured fungus	7.2	5.7	4.3	5.6*	
Hemicellulose	*Acidobacteria*	5.0	3.6*	3.1	3.7	
	*Proteobacteria*	18.1	15.0	12.5	10.0*	
	*Verrucomicrobia*	17.0	14.4	14.9	11.8*	
Lignin	*Proteobacteria*	8.1	6.2	6.1	4.0*	
	Unculturedbacterium	19.4	14.4	16.4	10.1*	
	*Ascomycota*	29.0	24.2**	21.5	21.9	
	*Basidiomycota*	101.6	83.9**	74.0	64.0	
	Uncultured fungus	13.8	11.3*	10.1	8.9	
Pectin	*Proteobacteria*	1.4	0.5*	0.4	0.2	
Chitin	*Actinobacteria*	84.0	71.6	66.9	53.0**	
	*Bacteroidetes*	21.2	18.4*	19.7	18.5	
	*Proteobacteria*	89.8	78.4*	80.7	75.8	
*Nitrogen cycling*						
Ammonification	*Actinobacteria*	62.8	59.4	45.1	37.1*	
	Unculturedbacterium	4.2	3.0*	3.4	2.0	
DNRA^b^	*Proteobacteria*	74.7	73.3	67.1	51.9*	
ANRA^c^	*Cyanobacteria*	9.3	11.9*	6.6	7.2	
	Unculturedbacterium	22.1	15.5**	14.7	9.7**	
Denitrification	*Proteobacteria*	53.6	49.2	37.8	28.0*	
	Unculturedbacterium	586.8	525.0*	451.1	388.2*	
Nitrogen fixation	*Chlorobi*	2.8	1.0*	0.6	0.6	
	*Firmicutes*	4.5	4.9	5.2	6.7*	
	*Proteobacteria*	38.8	34.9	26.2	16.9**	
	Unculturedbacterium	118.9	95.0**	97.0	85.3*	
*Phosphorus cycling*						
Phytase	*Ascomycota*	8.6	8.9	8.4	6.0*	
Exopolyphosphatase	*Actinobacteria*	20.0	16.4*	15.6	11.8*	
	*Bacteroidetes*	3.2	1.1	1.0	0.4*	
	*Cyanobacteria*	17.2	13.1	9.4	7.9**	

a The sum of normalized signal intensity for all probes detected within the same phylum. The t test was performed between the control- and the EcO157-inoculated biofilm (n = 3) at each sampling time and the significance was labeled at 24-S or 48-S (*P<0.1 and **P<0.05);

b DNRA, dissimilatory nitrate reduction;

c ANRA: assimilatory nitrate reduction. Detailed information regarding all phyla detected for genes involved in carbon degradation, nitrogen cycling and phosphorus utilization are presented in Tables S5, S6, and S7, respectively.

#### Cellulose

Hydrolysis of cellulose requires the concerted activity of endoglucanase, exoglucanase, and cellobiase. The abundance of the exoglucanase gene declined significantly in EcO157-inoculated biofilms at 24 h, whereas the abundance of the cellobiase gene decreased significantly at both 24 h and 48 h (Figure 4, Cellulose). Of the 180 DNA probes detecting exoglucanase or cellobiase, approximately half of them were from bacteria and the other half were from fungi. In the EcO157-inoculated biofilms, *Firmicutes* decreased significantly at 24 h only, whereas *Actinobacteria* decreased significantly at both sampling times (Table 3, Cellulose). In contrast, the abundance of the uncultured fungi increased significantly in 48-S biofilms compared with 48-C, but not for *Ascomycota* or *Basidiomycota* (Table S5).

#### Hemicellulose

DNA probes for hemicellulose degradation included 204, 64, 59, 131, and 60 for genes encoding the bacterial arabinofuranosidase, the fungal arabinofuranosidase, mannanase, xylose isomerase, and xylanase, respectively. Significant decreases in the mannanase- and bacterial arabinofuranosidase gene abundances were observed in EcO157-inoculated biofilms at 24 h and 48 h, respectively (Figure 4, Hemicellulose). Regardless of the high mannanase- and bacterial arabinofuranosidase gene abundances represented by *Actinobacteria* (31%), *Firmicutes* (23%), and *Bacteroidetes* (14%), they were not affected by the presence of EcO157 (Table S5). Rather, the abundances of DNA probes from *Acidobacteria*, *Proteobacteria*, or *Verrucomicrobia* decreased significantly in EcO157-inoculated biofilms at either 24 h or 48 h (Table 3).

#### Pectin

Among the 34 probes targeting the pectinase gene, a majority of the probes (∼70%) were from *Ascomycota* (*Aspergillus* spp. or *Penicillium* spp.). The presence of EcO157 did not have any dramatic impact on the abundance of fungal members; however, the abundance of *Proteobacteria* decreased significantly at 24 h (Table 3).

#### Lignin

Among the four lignin-degrading genes detected in spinach control biofilms, the abundance of the lignin peroxidase gene, the Mn-peroxidase gene, and the phenol oxidase gene declined significantly in EcO157-inoculated biofilms at 24 h (Figure 4, Lignin). Similarly to the pectin-degrading community, the majority of DNA probes representing the lignin-degrading community were from fungi, including *Basidiomycota* (∼57%), *Ascomycota* (∼16%), and uncultured fungi (10%). The abundances of all three fungal groups decreased significantly in EcO157-inoculated biofilms at 24 h, whereas at 48 h, the abundance of DNA probes from *Proteobacteria* and a group of uncultured bacteria declined significantly in EcO157-inoculated biofilms compared with the control (Table 3, Lignin).

#### Chitin

Genes encoding acetylglucosaminidase, endochitinase, and exochitinase were detected in control biofilms. In EcO157-inoculated biofilms, the abundance of the acetylglucosaminidase gene decreased significantly only at 24 h, and the abundance of the endochitinase gene decreased significantly at both 24 h and 48 h (Figure 4, Chitin). DNA probes targeting acetylglucosaminidase and endochitinase were primarily from four bacterial phyla and two fungal phyla (Table S5, Chitin). The presence of EcO157 appeared to have a major effect at 24 h on both *Bacteroidetes* and *Proteobacteria*, but, at 48 h, on *Actinobacteria* only (Table 3, Chitin).

### Nitrogen

**Figure 5:**
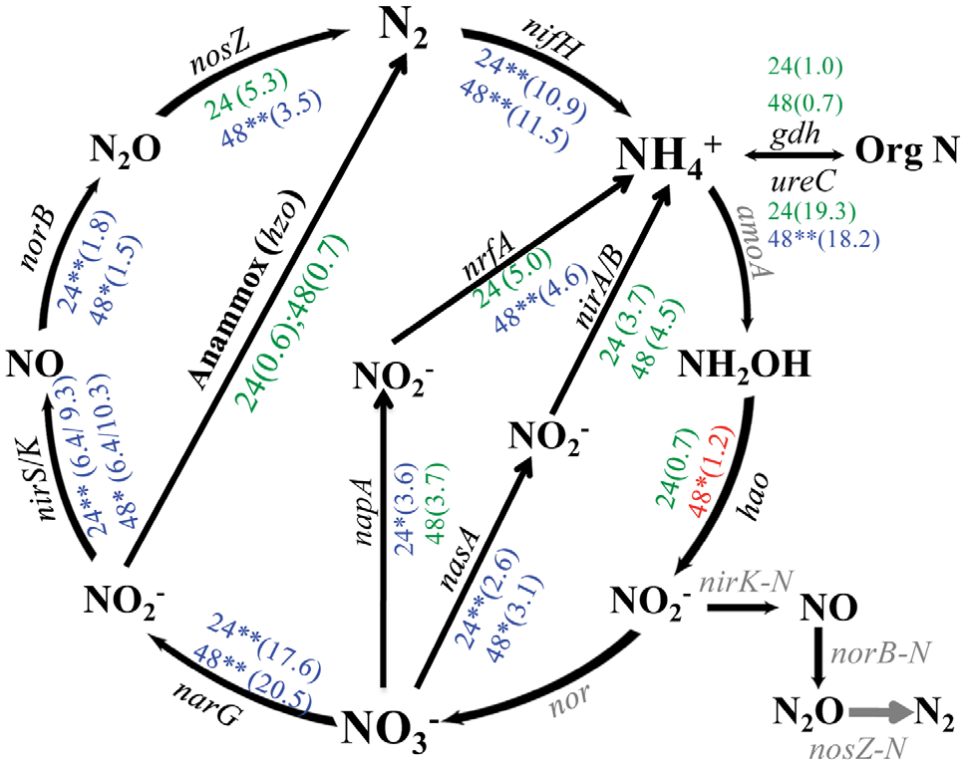
The impact of EcO157 on genes involved in nitrogen cycling in biofilm communities. The relative abundance of each gene was normalized to the total hybridization signals of all detected genes involved in nitrogen cycling. Genes labeled in black were detected by GeoChip whereas genes labeled in grey were either not detected by hybridization or not present on the GeoChip. The number 24, and 48 represents the biofilms at 24 h and 48 h, respectively. The number in a bracket followed either 24 or 48 was the sum of signal intensities of all detected gene probes divided by the sum of signal intensities of the detected nitrogen cycle genes, and weighted by the fold change of the signal intensities for this gene in EcO157-inoculated biofilms (24-S or 48-S) compared with controls (24-C or 48-C). For the number color corresponding to each gene, red designates that this gene had higher signal intensity in EcO157-inoculated biofilms than in the control biofilms; blue illustrates that this gene had lower signal intensity in EcO157-inoculated biofilms than in the control biofilms; and green designates no significant change in gene abundance between the control- and the EcO157-inoculated biofilms. The significance is indicated by either *(t test, *P*<0.1) or **(t test, *P*<0.05).

12 genes were detected in control biofilm communities. Most of those encode enzymes transforming various forms of nitrogen to ammonium (NH_4_^+^), such as genes involved in converting organic N into NH_4_^+^ through ammonification; converting nitrate to NH_4_^+^ through either dissimilarity nitrate reduction pathway (DNRA) or assimilatory nitrate reduction pathway (ANRA); transforming nitrate to nitrogen by denitrification; and the gene responsible for nitrogen reduction to NH_4_^+^. The presence of EcO157 led to a significant decrease in the abundance of nine genes either at 24 h only (*napA*), at 48 h only (*nrfA*, *nosZ*, and *ureC*), or at both sampling times (*nasA*, *narG*, *nirS/K*, *norB*, and *nifH*) (Figure 5, t test, *P*<0.1). In contrast, only one of three genes (*hao*) involved in nitrification (convert NH_4_^+^ to nitrate) was detected in control spinach biofilms. The abundance of *hao* in the EcO157-inoculated bioiflm was increased significantly only at 48 h (Figure 5 t test, *P*<0.1).

#### Ammonification

A total of 372 *ureC* gene probes from diverse phylo-groups, but predominantly from the *Proteobacteria* (∼60%) and *Actinobacteria* (26%), were detected in control biofilms (Table S6). The presence of EcO157 caused a significant decline of uncultured bacteria and *Actinobacteria* at 24- and 48 h, respectively (Table 3, Ammonification) but did not affect the abundance of the *Proteobacteria*.

#### Nitrate reduction

For DNRA pathway, all detected gene probes were from bacteria (Table S6). *Proteobacteria* represented the most probes (59%), and their abundance decreased significantly in the EcO157-inoculated biofilm at 48 h, whereas no change was observed for any other phylo-groups (Table 3, DNRA). For ANRA, the gene probes were distributed among *Cyanobacteria*, *Proteobacteria*, and a group of uncultured bacteria. In mixed biofilms with EcO157, the signal intensity of *nasA* probes from uncultured bacteria decreased significantly, but not for the *Proteobacteria* (Table 3, ANRA). Interestingly, at 24 h, the signal intensity of *Cyanobacteria nasA* increased almost 30% in the EcO157-inoculated biofilm, suggesting a positive interaction between the EcO157 and *Cyanobacteria* (Table 3, ANRA).

#### Denitrification

Over 1,000 probes representing denitrification were detected in control biofilms, with a majority from the uncultured bacteria (>80%) and about 10% from *Proteobacteia*. In mixed biofilms with EcO157, the abundance of the uncultured bacteria declined significantly at both 24- and 48 h, whereas the abundance of the *Proteobacteria* decreased significantly at 48 h only.

#### Nitrogen fixation

The *nifH* gene probes were primarily from *Proteobacteria* (∼21%) and uncultured bacteria (∼65%). Significant decreases in *nif*H intensity were observed for uncultured bacteria at both 24 h and 48 h, for *Chlorobi* at 24 h only, and for *Proteobacteria* at 48 h only (Table 3, Nitrogen fixation). Notably, at 48 h, the abundance of *Firmicutes nifH* increased about 28.8% in EcO157-inoculated biofilms, a trend observed for the *Cyanobacteria nasA* in the ANRA pathway.

### Phosphorus

In mixed biofilms with EcO157, a significant decrease in abundance of exopolyphosphatase gene (*ppx*) probes was observed at both sampling times, and for phytase, at 48 h only (Figure S3). However, there was no significant change in polyphosphate kinase gene abundance between control- and the EcO157-inoculated biofilms. For phytase, DNA probes from *Ascomycota* decreased significantly in 48-S biofilms, but not for those from *Basidiomycota*, or *Proteobacteria* (Table S7). Despite the high abundance of the *Proteobacteria ppx* gene, they appeared not to be affected by EcO157. Rather, the abundance of *ppx* probes from *Actinobacteria*, *Cyanobacteria*, and *Bacteroidetes* decreased significantly either over time or at 48 h only in the EcO157-inoculated biofilm (Table 3, Exopolyphosphatase).

## Discussion

As the occurrence of outbreaks of EcO157 infection linked to leafy vegetables is persisting, questions abound about the factors that affect its behavior in the production environment and lead to human disease. Harvesting and processing of leafy vegetables, such as spinach, inherently injures plant tissue and causes leakage of cell contents onto tools and equipment where biofilms may form. Our results demonstrate that EcO157 multiplies rapidly in fresh spinach lysates, with a growth rate comparable to that in rich culture broth such as LB. Spinach leaves contain an array of macronutrients and minerals that may nutritionally overlap the primary habitat of enteric bacteria, where simple sugars and host metabolites are the major resources. As a highly adapted enteric bacterium, *E. coli* is equipped with various transport proteins and pathways to uptake such nutrients efficiently [21], [22]. In contrast, many epiphytic bacteria have evolved to use nutritional resources economically with a tradeoff of slow growth known as altruistic strategy [23]. Indeed, three common successful phyllosphere colonists that were isolated from spinach leaves in our laboratory and identified by 16S rDNA sequencing as *Pseudomonas fluorescens*, *Pantoea agglomerans*, and *Xanthomonas campestris*, showed generation times of 106-, 80-, and 124 min, respectively, in full strength filter-sterilized spinach lysates. Under the same growth conditions, EcO157 had a considerably shorter generation time (42 min), suggesting that EcO157 has a greater ability to assimilate the substrates present in spinach leaf tissue.

Many epiphytic bacteria, including *Pseudomonas* spp., are proficient in biofilm formation. The synergistic interaction between *E. coli* and *P. putida* facilitates surface adhesion [24], and enhanced EcO157 biofilm formation by *P. aeruginosa* [25] have been reported previously. In our system, the spinach microorgansims enhanced the initial surface attachment of EcO157 to stainless steel, suggesting a potential mechanism of retention and transmission of EcO157 during produce production and processing. However, an antagonistic effect of the spinach microorgansims on EcO157 biofilm populations was observed at the later stage of biofilm formation. Spinach isolates that share a similar carbon utilization profile as that of EcO157 have been reported, as have been isolates producing compounds inhibitory to EcO157 [26]. Therefore, the inhibitory effect observed in our system could be the direct consequence of competition for substrates between EcO157 and other biofilm species, analogous to competition between EcO157 and *Enterobacter asburiae* reported previously [27], or due to secondary inhibition from toxic metabolites secreted by other biofilm members.

To gain a better understanding of the microbial dynamics in the spinach biofilm, we characterized its community with the GeoChip 4.0. Almost 27% of the DNA probes deposited on the GeoChip were detected in the spinach biofilm, suggesting a rich and diverse community. These DNA probes represent a wide range of functional categories, indicating also a high metabolic diversity of the biofilm. The *gyrB*-gene based phylogeny revealed members from more than 10 bacterial phyla, among which, the majority were α- and γ *Proteobacteria*, followed by *Actinobacteria, Firmicutes*, and *Bacteroidetes*. This phylogenetic composition is consistent with a previous report of spinach leaf surface bacterial communities measured by pyrosequencing [28], with the exception that we additionally observed relatively high abundances of *Cyanobacteria*, *Tenericutes*, *Verrucomicrobia*, and *Chlorobi*. This may stem from variations in phyllosphere microbial communities [9], [29], or from differences between the biofilm communities developed in spinach lysates and epiphytic consortia on spinach leaf surfaces, which would provide less abundant and diverse nutrients. This high diversity in the biofilm implies a robust microbial consortium capable of coping with environmental disturbances.

Examination of phylogeny origins of other DNA probes revealed the phylogenetic structure of various functional communities, such as carbon-, nitrogen- or phosphorus cycling communities. Similarly to the high abundance of chitin degradation gene probes from the γ-*Proteobacteria* and *Bacterioidetes* in our system, these two groups have been observed previously as the dominant members of a marine biofilm community that degrades insoluble polysaccharides [30]. Further, a large population of fungi (mainly *Ascomycota* and *Basidiomycota*) in the spinach biofilm appeared to play a key role in recycling nutrients by converting organic carbon or -phosphorus to bio-available simple carbohydrates or phosphorus. Biofilm formation by *Salmonella* on *Aspergillus niger* [31] and by *Pseudomonas aeruginosa* on *Candida albicans* [32] have been reported, suggesting a putative role for fungi in mixed biofilms that harbor human pathogens.

Analysis of the community composition in the biofilms over time in the absence and presence of EcO157 allowed for an assessment of the community-wide response to the human pathogen in the biofilm in spinach lysates. The decrease in community α-diversity of the biofilm in the presence of EcO157 suggested its strong antagonistic effect on certain microbial species, whereas the decrease in community β-diversity of EcO157-colonized biofilms at 48 h suggested a convergent shift in gene composition during biofilm maturation. Detailed analysis of functional genes affected by the presence of EcO157 supports the hypothesis that competition for nutrients is the primary mechanism of interaction. Among 24 genes detected and involved in converting complex carbon to simple sugars, 13 genes changed in abundance significantly in EcO157-inoculated biofilms. Similarly, among 14 genes involved in nitrogen transformation, 9 genes changed significantly in abundance, all of which contribute to the ammonia pool either directly or indirectly. We speculate that the rapid multiplication of EcO157 in spinach lysates leads to the bloom of the pathogen population along with the rapid depletion of easily assimilated nutrients in the biofilm. Such shift in metabolism was observed previously during development of a natural biofilm by meta-proteomic analysis [33]. Hence, at later stages of biofilm formation, abundance of ready-to-use carbon or ammonium may be low, resulting in increased competition from organisms capable of converting non-available carbon or nitrogen to their bio-available forms. Based on the GeoChip probes, EcO157 competed for carbon mainly with members of *Actinobacteria, Proteobacteria*, *Basidiomycota,* and uncultured fungi. For nitrogen, EcO157 appeared to compete primarily with *Proteobacteria* capable of converting nitrate to ammonia via the DNRA pathway, with members of uncultured bacteria that can transform nitrate to nitrogen by denitrification or nitrogen gas to ammonia by nitrogen fixation, and with *Actinobacteria* that can convert organic nitrogen to ammonia.

It is likely also, that competition selected for spinach epiphytes that metabolize resources not assimilated by EcO157 directly. Evidence for this is provided by the significant increase in abundance of *Cyanobacteria* capable of converting nitrate to ammonia via the ANRA pathway at 24 h, of the biofilm community members harboring the *hao* gene (mainly *Nitrosomonas* spp. and uncultured bacteria) at 48 h, and of fungal cellulose degradation genes and the *Firmicute*s *nifH* gene at 48 h in the EcO157-colonized biofilm, indicating a putative selection for these bacterial and fungal members carrying such functions in the community.

The interactions between EcO157 and indigenous spinach-associated microorganisms characterized in this study provide insight into both biotic and abiotic factors contributing to the persistence of EcO157 in produce processing environments. The metabolic potential of EcO157 in utilizing nutrients leaked from plant cells may contribute to the fitness of the human pathogen in plant-related environments. In turn, the epiphytic community, which is proficient in surface attachment, may enhance the biofilm capability of EcO157. The spinach biofilm community in our system was rich and diverse both phylogenetically and metabolically, suggesting considerable community resilience. Further characterization of microbial dynamics in mixed EcO157-indigenous microorgansims biofilms may provide clues for the control of human pathogens in crop and food production environments.

## Supporting Information

Figure S1 **Functional compositions of biofilm communities.**
(PDF)

Figure S2 **The gyrB-based phylo-composition of the biofilm community.**
(PDF)

Figure S3 **The impacts of EcO157 on the genes involved in phosphorus utilization in biofilm community.**
(PDF)

Table S1 **ANOVA test of EcO157 biofilm population sizes in various spinach lysates.**
(PDF)

Table S2 **Functional composition of biofilm communities detected with the GeoChip 4.0.**
(PDF)

Table S3 **The gyrB-based phylo-composition of biofilm communities.**
(PDF)

Table S4 **All genes with significant difference in signal intensity between the control- and the EcO157-inoculated biofilm community.**
(PDF)

Table S5 **Phylogeny origins of carbon degrading genes in biofilm communities.**
(PDF)

Table S6 **Phylogeny origins of genes involved in nitrogen cycling in biofilm communities.**
(PDF)

Table S7 **Phylogeny origins of genes involved in phosphorus utilization in biofilm communities.**
(PDF)
