# Supplementary material for: Functional Metagenomics of Escherichia coli O157:H7 Interactions with Spinach Indigenous Microorganisms during Biofilm Formation
Source: PLoS One. 2012 Sep 5;7(9):e44186. doi: 10.1371/journal.pone.0044186 (PMC3434221; doi:10.1371/journal.pone.0044186)
Supplement: Figure S1 — Functional compositions of biofilm communities. (PDF) [file pone.0044186.s001.pdf]

Figure S1. **Functional compositions of biofilm communities**. The relative abundance of each functional gene group was expressed as the percentage of signal intensity to the total in both biofilm communities (sum of 24-C and 48-C). (**A**) antibiotic resistance; (**B**) metal resistance; (**C**) stress-related; (**D**) virulence-related; (**E**) energy process; (**F**) carbon cycling; (**G**) nitrogen cycling; (**H**) phosphorus cycling; (**I**) sulphur utilization; (**J**) organic remediation; (**K**) phage related.

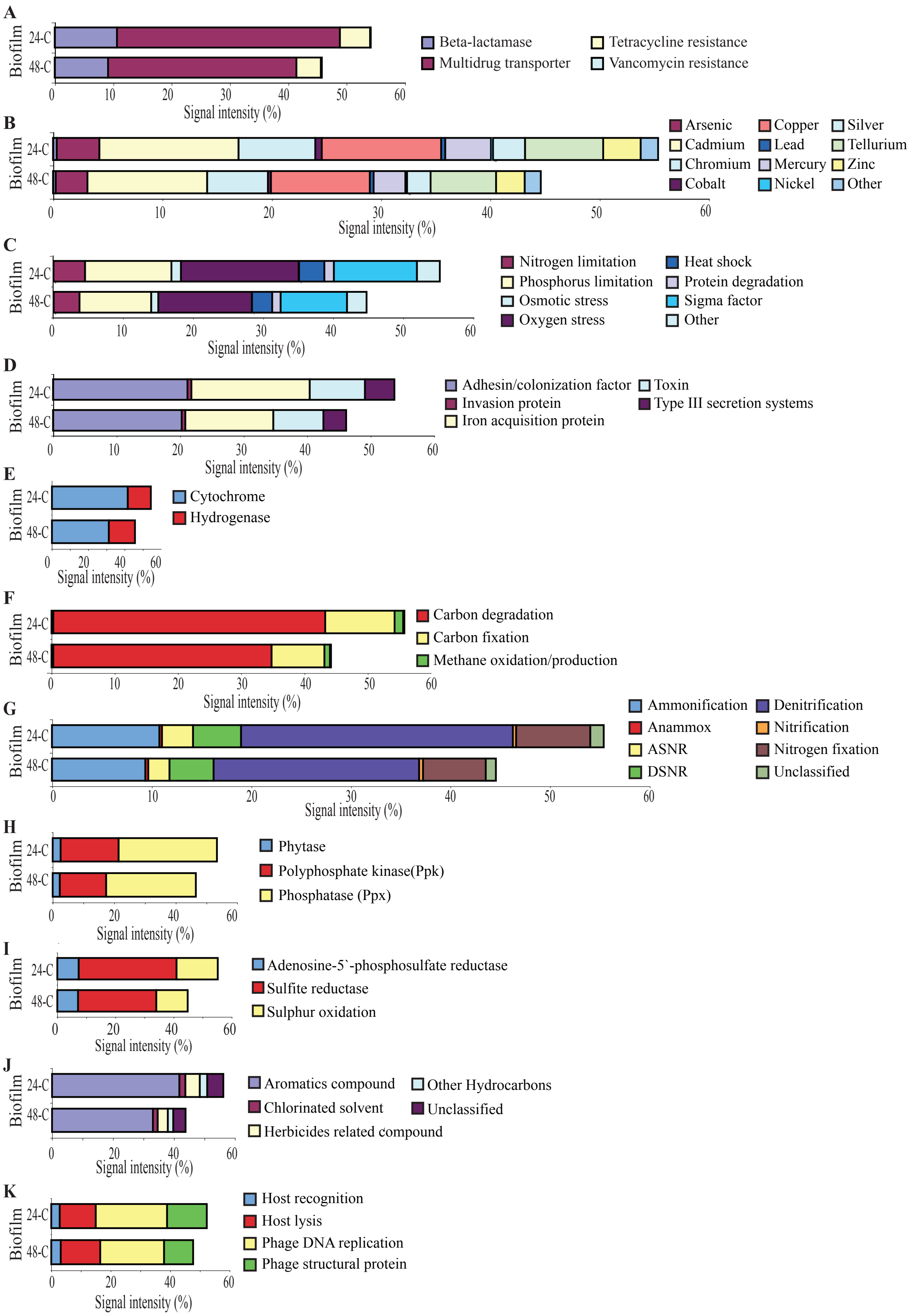
A
Biofilm
24-C
48-C
Beta-lactamase
Multidrug transporter
Tetracycline resistance
Vancomycin resistance
Signal intensity (%)
B
Arsenic
Cadmium
Chromium
Cobalt
Copper
Lead
Mercury
Nickel
Silver
Tellurium
Zinc
Other
C
Nitrogen limitation
Phosphorus limitation
Osmotic stress
Oxygen stress
Heat shock
Protein degradation
Sigma factor
Other
D
Adhesin/colonization factor
Invasion protein
Iron acquisition protein
Toxin
Type III secretion systems
E
Cytochrome
Hydrogenase
F
Carbon degradation
Carbon fixation
Methane oxidation/production
G
Ammonification
Anammox
ASNR
DSNR
Denitrification
Nitrification
Nitrogen fixation
Unclassified
H
Phytase
Polyphosphate kinase(Ppk)
Phosphatase (Ppx)
I
Adenosine-5`-phosphosulfate reductase
Sulfite reductase
Sulphur oxidation
J
Aromatics compound
Chlorinated solvent
Herbicides related compound
Other Hydrocarbons
Unclassified
K
Host recognition
Host lysis
Phage DNA replication
Phage structural protein

Figure S1
