# Supplementary material for: Functional Metagenomics of Escherichia coli O157:H7 Interactions with Spinach Indigenous Microorganisms during Biofilm Formation
Source: PLoS One. 2012 Sep 5;7(9):e44186. doi: 10.1371/journal.pone.0044186 (PMC3434221; doi:10.1371/journal.pone.0044186)
Supplement: Figure S2 — The gyrB-based phylo-composition of the biofilm community. (PDF) [file pone.0044186.s002.pdf]

Figure S2. The *gyrB*-based phylo-composition of the biofilm community. The phylo-composition of biofilm was examined at the phylum level except for *proteobacteria*, which was expressed at the class level. The total abundance of each phylum was represented by the sum of the normalized signal intensities of all *gryB* probes detected in the same phylum or class. Black columns represent the biofilm community at 24 h (24-C), whereas white columns represent the biofilm community at 48 h (48-C). The significance is indicated by either * (t test, $P<0.1$) or ** (t test, $P<0.05$).

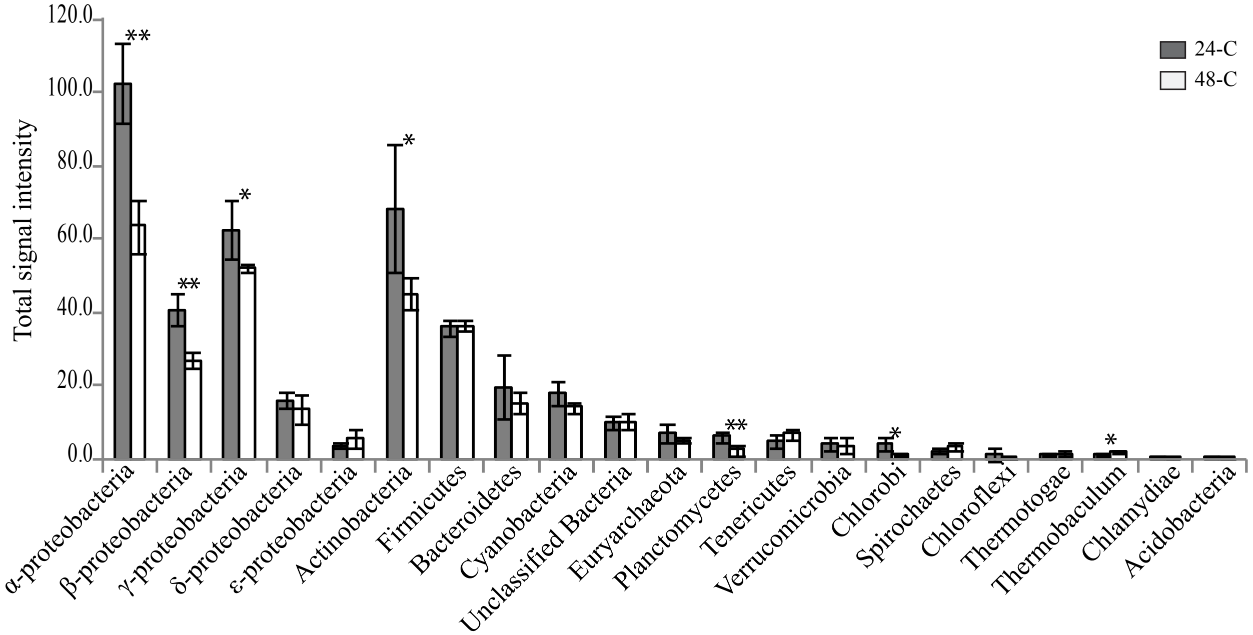

Total signal intensity
120.0
100.0
80.0
60.0
40.0
20.0
0.0
**
**
*
*
**
*
*
24-C
48-C
α-proteobacteria
β-proteobacteria
γ-proteobacteria
δ-proteobacteria
ε-proteobacteria
Actinobacteria
Firmicutes
Bacteroidetes
Cyanobacteria
Unclassified Bacteria
Euryarchaeota
Planctomycetes
Tenericutes
Verrucomicrobia
Chlorobi
Spirochaetes
Chloroflexi
Thermotogae
Thermobaculum
Chlamydiae
Acidobacteria

Figure S2
