# Supplementary material for: Functional Metagenomics of Escherichia coli O157:H7 Interactions with Spinach Indigenous Microorganisms during Biofilm Formation
Source: PLoS One. 2012 Sep 5;7(9):e44186. doi: 10.1371/journal.pone.0044186 (PMC3434221; doi:10.1371/journal.pone.0044186)
Supplement: Figure S3 — The impacts of EcO157 on the genes involved in phosphorus utilization in biofilm community. (PDF) [file pone.0044186.s003.pdf]

Figure S3. **The impacts of EcO157 on the genes involved in phosphorus utilization in biofilm community.** Panels A and B are biofilms at 24 h and 48 h, respectively. The abundance of each gene was the average signal intensity of the all gene probes detected for that gene in each biofilm community. The error bar represents the standard error for each gene. Genes with a significant decrease in the abundance between the control- and the EcO157-inoculated biofilm communities were marked with an asterisk (*) (t test, $P<0.05$).

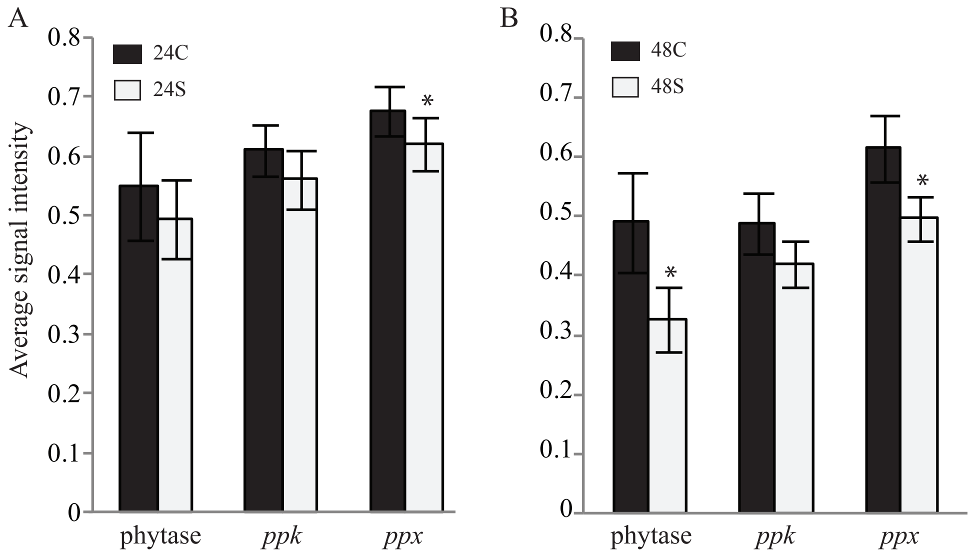
A
B
0.8
0.7
0.6
0.5
0.4
0.3
0.2
0.1
0
Average signal intensity
24C
24S
48C
48S
*
phytase
ppk
ppx

Figure S3
