# Supplementary material for: Functional Metagenomics of Escherichia coli O157:H7 Interactions with Spinach Indigenous Microorganisms during Biofilm Formation
Source: PLoS One. 2012 Sep 5;7(9):e44186. doi: 10.1371/journal.pone.0044186 (PMC3434221; doi:10.1371/journal.pone.0044186)
Supplement: Table S1 — ANOVA test of EcO157 biofilm population sizes in various spinach lysates. (PDF) [file pone.0044186.s004.pdf]

Table S1. ANOVA test of EcO157 biofilm population sizes in various spinach lysates

Time (h)	Pair-wise comparison			
	NS[a]100% vs 5%	NS100% vs 0.1%	NS100% vs S[b]100%	5% vs 0.1%
6	No	*P*<0.05	*P*<0.05	*P*<0.05
24	*P*<0.05	*P*<0.05	No	No
48	*P*<0.001	*P*<0.001	*P*<0.05	*P*<0.05

[a] NS represents non-sterile spinach lysates; [b]S represents sterile spinach lysates. 100% represents undiluted spinach lysates; 5%- and 0.1% are the percentage of non-sterile spinach lysates in sterile water.
