# Supplementary material for: Functional Metagenomics of Escherichia coli O157:H7 Interactions with Spinach Indigenous Microorganisms during Biofilm Formation
Source: PLoS One. 2012 Sep 5;7(9):e44186. doi: 10.1371/journal.pone.0044186 (PMC3434221; doi:10.1371/journal.pone.0044186)
Supplement: Table S2 — Functional composition of biofilm communities detected with the GeoChip 4.0. (PDF) [file pone.0044186.s005.pdf]

Table S2. Functional composition of biofilm communities detected with the GeoChip 4.0

Functional category	Number of detected gene probes	Sum of signal intensity[a]	
		24-C	48-C
Antibiotic resistance	1337		
Beta-lactamase	291	165.0	141.7
Multidrug transporter	922	592.7	500.2
Tetracycline resistance	118	79.4	64.5
Vancomycin resistance	6	2.3	3.2
Metal resistance	4011		
Aluminum	35	15.7	11.7
Arsenic	293	187.7	139.0
Cadmium	939	610.1	524.6
Chromium	464	335.7	265.5
Cobalt	29	28.0	14.5
Copper	749	523.1	433.5
Lead	31	18.3	15.3
Mercury	344	199.9	139.2
Nickel	19	8.8	7.4
Silver	218	141.6	103.1
Tellurium	511	341.5	287.3
Zinc	248	165.1	125.4
Other	131	75.0	70.3
Stress tolerance	7315		
Glucose limitation	28	10.6	13.4
Nitrogen limitation	588	370.2	301.6
Phosphorus limitation	1668	1016.2	843.6
Osmotic stress	165	112.2	82.9
Oxygen stress	2118	1383.0	1100.0
Cold shock	19	5.3	3.7
Heat shock	518	297.3	236.5
Protein degradation	188	111.3	98.8
Sigma factor	1555	978.0	781.8
Other	468	267.4	229.4
Virulence	1362		
Adhesin/colonization factor	574	330.5	316.2
Invasion protein	24	9.7	8.7
Iron acquisition protein	395	291.0	216.5
Toxin	248	135.5	123.1
Type III secretion systems	121	72.2	56.1
Carbon cycling	4207		
Acetogenesis	33	13.3	12.5
Carbon degradation	3267	1994.1	1600.8
Carbon fixation	732	508.1	387.9
Methane oxidation and production	156	62.5	41.6
Others	19	8.8	6.8
Nitrogen cycling	2452		
Ammonification	378	265.3	231.0
Anerobic ammonium oxidation	10	6.7	6.5
Assimilatory nitrogen reduction	133	76.2	52.9
Dissimilatory nitrogen reduction	209	118.7	108.7
Denitrification	1180	670.4	507.5
Nitrification	18	8.1	9.4
Nitrogen fixation	462	183.5	155.0
Unclassified	65	33.2	25.2

Phosphorus utilization	520		
Phytase	31	17.1	15.2
Polyphosphate kinase(Ppk)	190	123.4	98.8
Phosphatase (Ppx)	299	209.9	191.3
Sulphur utilization	1185		
Adenosine-5`-phosphosulfate reductase	165	86.7	84.2
Sulfite reductase	799	396.1	316.5
Sulphur oxidation	221	166.8	127.0
Energy process	378		
Cytochrome	290	198.1	148.5
Hydrogenase	88	60.0	68.4
Organic degradation	8102		
Aromatic compound	5946	4003.5	3169.0
Chlorinated solvent	261	187.7	153.2
Herbicide related compound	687	450.5	316.6
Other Hydrocarbons	368	233.6	172.7
Unclassified	840	506.9	380.8
Phage-related gene	229		
Host recognition	11	5.7	6.4
Host lysis	43	24.3	26.7
Phage DNA replication	123	48.2	43.2
Phage structural protein	52	26.8	19.6

[a]The sum of normalized signal intensity for the all probes detected within the same functional category.
