# Supplementary material for: Functional Metagenomics of Escherichia coli O157:H7 Interactions with Spinach Indigenous Microorganisms during Biofilm Formation
Source: PLoS One. 2012 Sep 5;7(9):e44186. doi: 10.1371/journal.pone.0044186 (PMC3434221; doi:10.1371/journal.pone.0044186)
Supplement: Table S3 — The gyrB-based phylo-composition of biofilm communities. (PDF) [file pone.0044186.s006.pdf]

Table S3. The *gyrB*-based phylo-composition of biofilm communities

Phylum	Sum of signal intensity[a]	
	24-C	48-C
Proteobacteria	226.1 ± 54.8	162.6 ± 52.1
α-proteobacteria	102.6 ± 10.9	63.7 ± 7.4
β-proteobacteria	40.9 ± 4.5	27.1 ± 2.4
γ-proteobacteria	62.8 ± 7.8	52.1 ± 1.1
δ-proteobacteria	16.0 ± 2.2	13.7 ± 3.9
ε-proteobacteria	3.9 ± 0.5	6.0 ± 2.6
Actinobacteria	68.6 ± 17.5	45.3 ± 4.7
Firmicutes	36.1 ± 2.3	36.4 ± 1.5
Bacteroidetes	19.8 ± 8.6	15.2 ± 2.9
Cyanobacteria	18.3 ± 3.2	14.2 ± 1.5
Unclassified Bacteria	10.0 ± 1.7	10.4 ± 2.1
Euryarchaeota	7.1 ± 2.4	5.1 ± 0.7
Planctomycetes	6.1 ± 1.5	2.6 ± 1.4
Tenericutes	5.0 ± 1.5	6.9 ± 1.4
Verrucomicrobia	4.4 ± 2.0	3.7 ± 2.3
Chlorobi	4.3 ± 1.7	1.6 ± 0.3
Spirochaetes	2.4 ± 0.7	3.6 ± 1.2
Chloroflexi	1.3 ± 1.8	0.3 ± 0.2
Thermotogae	1.3 ± 0.1	1.6 ± 0.7
Thermobaculum	1.0 ± 0.4	1.9 ± 0.3
Chlamydiae	0.5 ± 0.5	0.2 ± 0.3
Acidobacteria	0.4 ± 0.3	0.3 ± 0.2

[a]The sum of normalized signal intensity for the all probes detected within the same phylum or class.
