# Supplementary material for: Functional Metagenomics of Escherichia coli O157:H7 Interactions with Spinach Indigenous Microorganisms during Biofilm Formation
Source: PLoS One. 2012 Sep 5;7(9):e44186. doi: 10.1371/journal.pone.0044186 (PMC3434221; doi:10.1371/journal.pone.0044186)
Supplement: Table S4 — All genes with significant difference in signal intensity between the control- and the EcO157-inoculated biofilm community. (PDF) [file pone.0044186.s007.pdf]

Table S4. All genes with significant difference in signal intensity between the control- and the EcO157-inoculated biofilm community

Gene name or protein	Gene category	Sub-category	Biofilms[a]
B_lactamase_A	Antibiotic resistance	Beta-lactamases	24-C and 24-S
Tet	Antibiotic resistance	other	48-C and 48-S
lysin	Bacteria phage	LYSIS	24-C and 24-S
uvsW	Bacteria phage	REPLICATION	24-C and 24-S
DNA_polymerase_type_I	Bacteria phage	REPLICATION	48-C and 48-S
primase	Bacteria phage	REPLICATION	48-C and 48-S
T4_type_portal_protein	Bacteria phage	REPLICATION	48-C and 48-S
scaffold	Bacteria phage	STRUCTURAL	24-C and 24-S
FTHFS	Carbon cycling	Acetogenesis	48-C and 48-S
cellobiase	Carbon cycling	Carbon degradataion	24-C and 24-S; 48-C and 48-S
exoglucanase	Carbon cycling	Carbon degradataion	24-C and 24-S
endochitinase	Carbon cycling	Carbon degradataion	24-C and 24-S
phenol_oxidase	Carbon cycling	Carbon degradataion	24-C and 24-S
glucoamylase	Carbon cycling	Carbon degradataion	24-C and 24-S
vanA	Carbon cycling	Carbon degradataion	48-C and 48-S
amyA	Carbon cycling	Carbon degradataion	48-C and 48-S
pulA	Carbon cycling	Carbon degradataion	48-C and 48-S
rubisco	Carbon cycling	Carbon fixation	24-C and 24-S
pcc	Carbon cycling	Carbon fixation	24-C and 24-S
aclB	Carbon cycling	Carbon fixation	24-C and 24-S
CODH	Carbon cycling	Carbon fixation	48-C and 48-S
mmoX	Carbon cycling	Methane	24-C and 24-S
mcrA	Carbon cycling	Methane	24-C and 24-S
P450	Energy process	NA	24-C and 24-S
Ni_Fe_hydrogenase	Energy process	NA	24-C and 24-S
cytochrome	Energy process	Energy process	24-C and 24-S
Al	Metal Resistance	Aluminum	48-C and 48-S
ArsA	Metal Resistance	Arsenic	24-C and 24-S; 48-C and 48-S
ArsC	Metal Resistance	Arsenic	24-C and 24-S
CadA	Metal Resistance	Cadmium	24-C and 24-S
czcA	Metal Resistance	Cadmium,Cobalt,Zinc	24-C and 24-S
czcC	Metal Resistance	Cadmium,Cobalt,Zinc	48-C and 48-S
ChrA	Metal Resistance	Chromium	24-C and 24-S
CopA	Metal Resistance	Copper	24-C and 24-S
CusF	Metal Resistance	Copper	24-C and 24-S
pbrD	Metal Resistance	Lead	24-C and 24-S
pbrT	Metal Resistance	Lead	24-C and 24-S
mer	Metal Resistance	Mercury	24-C and 24-S
silC	Metal Resistance	Silver	24-C and 24-S
silA	Metal Resistance	Silver	48-C and 48-S
terC	Metal Resistance	Tellurium	24-C and 24-S; 48-C and 48-S
tehB	Metal Resistance	Tellurium	24-C and 24-S
zntA	Metal Resistance	Zinc	24-C and 24-S
nasA	Nitrogen	Assimilatory N reduction	48-C and 48-S
narG	Nitrogen	Denitrification	24-C and 24-S
nirS	Nitrogen	Denitrification	24-C and 24-S
napA	Nitrogen	Dissimilatory N reduction	24-C and 24-S
nifH	Nitrogen	Nitrogen fixation	24-C and 24-S; 48-C and 48-S
todF	Organic Remediation	NA	24-C and 24-S; 48-C and 48-S

catB	Organic Remediation	Aromatics	24-C and 24-S; 48-C and 48-S
tftH	Organic Remediation	Aromatics	24-C and 24-S; 48-C and 48-S
pobA	Organic Remediation	Aromatics	24-C and 24-S; 48-C and 48-S
phtA	Organic Remediation	Aromatics	24-C and 24-S; 48-C and 48-S
pimF	Organic Remediation	Aromatics	24-C and 24-S
nmoA	Organic Remediation	Aromatics	24-C and 24-S
bclA	Organic Remediation	Aromatics	24-C and 24-S
mdlC	Organic Remediation	Aromatics	24-C and 24-S
phaB	Organic Remediation	Aromatics	24-C and 24-S
oxdB	Organic Remediation	Aromatics	24-C and 24-S
catechol_B	Organic Remediation	Aromatics	24-C and 24-S
flnB	Organic Remediation	Aromatics	24-C and 24-S
nagG	Organic Remediation	Aromatics	24-C and 24-S
benAB	Organic Remediation	Aromatics	24-C and 24-S
hmgC	Organic Remediation	Aromatics	24-C and 24-S
apc	Organic Remediation	Aromatics	24-C and 24-S
bbs	Organic Remediation	Aromatics	24-C and 24-S
pchCF	Organic Remediation	Aromatics	24-C and 24-S
Arylest	Organic Remediation	Aromatics	24-C and 24-S
BADH	Organic Remediation	Aromatics	24-C and 24-S
Catechol	Organic Remediation	Aromatics	24-C and 24-S
tdnB	Organic Remediation	Aromatics	24-C and 24-S
nahA	Organic Remediation	Aromatics	24-C and 24-S
mhpB	Organic Remediation	Aromatics	48-C and 48-S
bbsG	Organic Remediation	Aromatics	48-C and 48-S
cbdA	Organic Remediation	Aromatics	48-C and 48-S
bphB	Organic Remediation	Aromatics	48-C and 48-S
arhA	Organic Remediation	Aromatics	48-C and 48-S
dxnA	Organic Remediation	Aromatics	48-C and 48-S
phdCI	Organic Remediation	Aromatics	48-C and 48-S
phdI	Organic Remediation	Aromatics	48-C and 48-S
trzN	Organic Remediation	Herbicides related	24-C and 24-S
phn	Organic Remediation	Herbicides related	48-C and 48-S
atzB	Organic Remediation	Herbicides related	48-C and 48-S
atzC	Organic Remediation	Herbicides related	48-C and 48-S
atzD	Organic Remediation	Herbicides related	48-C and 48-S
chnE	Organic Remediation	Other Hydrocarbons	24-C and 24-S
Xamo	Organic Remediation	Other Hydrocarbons	48-C and 48-S
alkH	Organic Remediation	Other Hydrocarbons	48-C and 48-S
alkJ	Organic Remediation	Other Hydrocarbons	48-C and 48-S
msmABCD	Organic Remediation	Others	24-C and 24-S; 48-C and 48-S
alkK	Organic Remediation	Others	24-C and 24-S
MSAD	Organic Remediation	Others	48-C and 48-S
linB	Organic Remediation	Pesticides related compound	24-C and 24-S; 48-C and 48-S
linC	Organic Remediation	Pesticides related compound	48-C and 48-S
gyrB	other category	Phylogenetic marker	24-C and 24-S
phytase	Phosphorus	Phosphorus utilization	24-C and 24-S; 48-C and 48-S
desR	Stress	Cold shock	24-C and 24-S
grpE	Stress	Heat shock	24-C and 24-S
glnA	Stress	Nitrogen limitation	48-C and 48-S
proV	Stress	Osmotic stress	24-C and 24-S
cydA	Stress	Oxygen limitation	48-C and 48-S

arcA	Stress	Oxygen limitation	48-C and 48-S
katE	Stress	Oxygen stress	24-C and 24-S
ahpC	Stress	Oxygen stress	24-C and 24-S
ahpF	Stress	Oxygen stress	48-C and 48-S
pstA	Stress	Phosphate limitation	24-C and 24-S
pstB	Stress	Phosphate limitation	24-C and 24-S
phoB	Stress	Phosphate limitation	24-C and 24-S
phoA	Stress	Phosphate limitation	24-C and 24-S
pstC	Stress	Phosphate limitation	48-C and 48-S
clpC	Stress	Protein stress	24-C and 24-S
sigma_24	Stress	Sigma factors	24-C and 24-S; 48-C and 48-S
sigma_70	Stress	Sigma factors	24-C and 24-S
rpoS (sigma 38)	Stress	Sigma factors	48-C and 48-S
APS_AprB	Sulphur	NA	24-C and 24-S
APS_AprA	Sulphur	NA	24-C and 24-S
aprA	Sulphur	Other	48-C and 48-S
dsrA	Sulphur	sulfite reductase	24-C and 24-S
dsrB	Sulphur	sulfite reductase	24-C and 24-S
iro	virulence	NA	24-C and 24-S
vip	virulence	NA	24-C and 24-S
cap	virulence	NA	24-C and 24-S

[a]The t test was performed between the control- and the EcO157 inoculated biofilm (n=3) at each time point (24 h or 48 h) using all detected probes for each gene. Significance was measured by $P<0.05$.
