# Supplementary material for: Functional Metagenomics of Escherichia coli O157:H7 Interactions with Spinach Indigenous Microorganisms during Biofilm Formation
Source: PLoS One. 2012 Sep 5;7(9):e44186. doi: 10.1371/journal.pone.0044186 (PMC3434221; doi:10.1371/journal.pone.0044186)
Supplement: Table S5 — Phylogeny origins of carbon degrading genes in biofilm communities. (PDF) [file pone.0044186.s008.pdf]

Table S5. Phylogeny origins of carbon degrading genes in biofilm communities

Functional group	Phylum[a]	Normalized signal intensity[b]			
		24-C	24-S	48-C	48-S
Starch degradation (α-amylase, glucoamylase, pullulanase)	*Euryarchaeota*	11.7 ± 1.1	12.8 ± 0.8	12.6 ± 1.7	13.6 ± 1.6
	Actinobacteria	104.1 ± 8.1	92.7 ± 12.0	89.0 ± 5.3	66.0 ± 6.4**
	Chloroflexi	12.1 ± 2.3	10.3 ± 0.6	8.2 ± 0.6	9.1 ± 0.8
	Cyanobacteria	10.3 ± 1.5	10.0 ± 0.7	9.2 ± 1.1	8.2 ± 1.8
	Firmicutes	30.8 ± 3.4	30.1 ± 2.1	29.7 ± 5.5	27.7 ± 4.9
	Proteobacteria	142.5 ± 15.5	130.1 ± 12.0	104.8 ± 17.0	90.9 ± 1.0
	Ascomycota	15.1 ± 1.4	14.8 ± 1.6	12.6 ± 1.3	12.6 ± 1.9
Cellulose degradation (Exoglucanase, cellobiase)	***Actinobacteria***	34.9 ± 4.6	27.1 ± 4.9*	24.5 ± 1.3	15.6 ± 1.8**
	Firmicutes	6.7 ± 0.9	5.2 ± 1.0*	6.9 ± 2.4	7.0 ± 2.0
	Proteobacteria;	20.1 ± 3.3	18.7 ± 3.4	13.2 ± 2.2	11.6 ± 1.1
	Ascomycota	28.6 ± 5.2	24.7 ± 3.3	18.2 ± 1.6	20.4 ± 0.5
	Basidiomycota	7.9 ± 2.4	7.5 ± 2.2	5.5 ± 0.9	7.1 ± 2.7
	**Uncultured fungus**	7.2 ± 0.6	5.7 ± 1.2	4.3 ± 0.5	5.6 ± 0.9*
Hemicellulose degradation (Bacterial arabinofuranosidase, mannanase)	***Acidobacteria***	5.0 ± 0.8	3.6 ± 0.9*	3.1 ± 0.2	3.7 ± 2.7
	Actinobacteria	40.1 ± 6.3	35.9 ± 4.1	39.9 ± 17.0	32.4 ± 4.3
	Bacteroidetes	18.5 ± 2.0	18.2 ± 0.7	19.6 ± 3.2	19.9 ± 0.3
	Firmicutes	30.0 ± 3.8	31.5 ± 2.6	35.6 ± 4.9	31.9 ± 3.0
	Proteobacteria	18.1 ± 3.2	15.0 ± 1.3	12.5 ± 1.2	10.0 ± 1.7*
	Verrucomicrobia	17.0 ± 0.5	14.4 ± 3.1	14.9 ± 0.8	11.8 ± 2.7*
Lignin degradation (lignin peroxidase, Mn peroxidase, phenol oxidase)	*Actinobacteria*	1.8 ± 0.5	2.3 ± 0.6	2.4 ± 0.7	3.2 ± 0.4
	Proteobacteria	8.1 ± 1.6	6.2 ± 1.4	6.1 ± 1.6	4.0 ± 0.8*
	**Uncultured bacterium**	19.4 ± 5.0	14.4 ± 1.1	16.4 ± 4.2	10.1 ± 2.2*
	Ascomycota	29.0 ± 2.1	24.2 ± 1.3**	21.5 ± 2.3	21.9 ± 1.4
	Basidiomycota	101.6 ± 6.0	83.9 ± 3.7**	74.0 ± 24.9	64.0 ± 3.4
	**Uncultured fungus**	13.8 ± 1.2	11.3 ± 2.1*	10.1 ± 1.0	8.9 ± 0.4
Pectin degradation (pectinase)	***Proteobacteria***	1.4 ± 0.7	0.5 ± 0.2*	0.4 ± 0.4	0.2 ± 0.3
	Uncultured bacterium	1.4 ± 0.6	1.3 ± 0.4	0.6 ± 0.4	0.3 ± 0.3
	Ascomycota	7.8 ± 2.3	7.0 ± 1.5	4.4 ± 0.9	4.6 ± 1.2
Chitin degradation (acetylglucosaminidase, endochitinase)	***Actinobacteria***	84.0 ± 13.8	71.6 ± 10.5	66.9 ± 5.6	53.0 ± 4.7**
	Bacteroidetes	21.2 ± 2.1	18.4 ± 1.7*	19.7 ± 2.1	18.5 ± 2.7
	Firmicutes	11.7 ± 1.8	11.5 ± 1.4	11.0 ± 5.3	11.9 ± 1.7
	Proteobacteria	89.8 ± 2.8	78.4 ± 8.7*	80.7 ± 18.3	75.8 ± 7.9
	Uncultured bacterium	18.2 ± 2.3	16.3 ± 2.5	12.1 ± 3.1	9.1 ± 2.1
	Ascomycota	48.5 ± 8.1	42.3 ± 3.7	32.2 ± 2.5	29.4 ± 4.7
	Basidiomycota	8.1 ± 3.7	5.7 ± 0.6	5.6 ± 1.2	6.0 ± 1.5

[a]The phyla in bold were those with a significant change in their abundance between the control- and the EcO157-inoculated biofilm at either 24 h or 48 h; [b]The sum of normalized signal intensity for the all probes detected within the same phylum. The t test was performed between the control- and the EcO157 inoculated biofilm (n=3) at each time point and the significance was labeled at 24-S or 48-S (* P<0.1 and ** P<0.05).
