# Supplementary material for: Functional Metagenomics of Escherichia coli O157:H7 Interactions with Spinach Indigenous Microorganisms during Biofilm Formation
Source: PLoS One. 2012 Sep 5;7(9):e44186. doi: 10.1371/journal.pone.0044186 (PMC3434221; doi:10.1371/journal.pone.0044186)
Supplement: Table S6 — Phylogeny origins of genes involved in nitrogen cycling in biofilm communities. (PDF) [file pone.0044186.s009.pdf]

Table S6. Phylogeny origins of genes involved in nitrogen cycling in biofilm communities

Nitrogen cycling process (gene)	Phylum[a]	Normalized signal intensity[b]			
		24-C	24-S	48-C	48-S
Ammonification (*ureC*)	***Actinobacteria***	62.8 ± 6.9	59.4 ± 17.0	45.1 ± 4.8	37.1 ± 5.4*
	Cyanobacteria	12.9 ± 1.3	13.3 ± 0.7	12.7 ± 2.1	14.0 ± 4.7
	Firmicutes	6.3 ± 1.6	7.9 ± 2.5	7.2 ± 1.1	10.3 ± 3.7
	Proteobacteria	146.4 ± 3.9	149.4 ± 8.0	138.2 ± 31.3	117.7 ± 5.2
	**Uncultured bacterium**	4.2 ± 0.7	3.0 ± 0.6*	3.4 ± 1.5	2.0 ± 1.0
	Ascomycota	10.9 ± 1.0	11.7 ± 2.5	9.5 ± 1.5	8.3 ± 1.4
Dissimilatory nitrogen reduction (*napA* and *nrfA*)	*Actinobacteria*	1.8 ± 0.6	1.2 ± 0.5	1.2 ± 0.4	0.4 ± 0.3
	Bacteroidetes	4.4 ± 0.5	5.2 ± 0.7	4.1 ± 0.9	3.9 ± 0.4
	Firmicutes	5.4 ± 1.4	4.9 ± 0.4	4.1 ± 1.0	3.7 ± 1.0
	Proteobacteria	74.7 ± 6.7	73.3 ± 6.1	67.1 ± 10.5	51.9 ± 9.5*
	Uncultured bacteria	32.6 ± 2.2	28.9 ± 3.5	32.4 ± 8.1	30.8 ± 2.1
	Verrucomicrobia	1.4 ± 0.7	1.5 ± 1.5	0.6 ± 0.1	0.8 ± 1.0
Assimilatory nitrogen reduction (*nasA*)	***Cyanobacteria***	9.3 ± 1.5	11.9 ± 1.7*	6.6 ± 0.8	7.2 ± 1.4
	Proteobacteria	19.2 ± 3.0	16.0 ± 2.6	11.1 ± 0.9	10.9 ± 0.6
	**Uncultured bacterium**	22.1 ± 2.6	15.5 ± 1.5**	14.7 ± 1.6	9.7 ± 1.6**
Denitrification (*narG*, *nirK/nirS*, *norB*, and *nosZ*)	*Actinobacteria*	4.2 ± 0.8	3.6 ± 0.6	2.3 ± 0.6	1.8 ± 0.2
	Proteobacteria	53.6 ± 10.7	49.2 ± 6.3	37.8 ± 7.3	28.0 ± 2.7*
	**Uncultured bacterium**	586.8 ± 53.5	525.0 ± 20.1*	451.1 ± 65.3	388.2 ± 16.0*
Nitrogen fixation (*nifH*)	*Euryarchaeota;*	9.4 ± 0.8	9.4 ± 1.2	10.7 ± 1.9	10.0 ± 1.4
	Actinobacteria	3.4 ± 0.8	3.7 ± 0.8	3.9 ± 1.5	4.6 ± 0.1
	**Chlorobi**	2.8 ± 1.2	1.0 ± 0.5*	0.6 ± 0.2	0.6 ± 0.4
	Cyanobacteria	4.8 ± 0.5	4.7 ± 0.2	4.2 ± 1.6	3.8 ± 0.5
	Firmicutes	4.5± 1.1	4.9 ± 1.4	5.2 ± 1.1	6.7 ± 0.3*
	Proteobacteria	38.8 ± 2.8	34.9 ± 6.5	26.2 ± 2.5	16.9 ± 1.2**
	**Uncultured bacterium**	118.9 ± 5.5	95.0 ± 1.7**	97.0 ± 7.2	85.3 ± 5.0*

[a]The phyla in bold were those with a significant change in their abundance between the control- and the EcO157-inoculated biofilm at either 24 h or 48 h; [b]The sum of normalized signal intensity for the all DNA probes detected within the same phylum. The t test was performed between the control- and pathogen inoculated biofilm (n=3) at each time point and the significance was labeled at 24-S or 48-S (* *P*<0.1 and ** *P*<0.05).
