# Supplementary material for: Functional Metagenomics of Escherichia coli O157:H7 Interactions with Spinach Indigenous Microorganisms during Biofilm Formation
Source: PLoS One. 2012 Sep 5;7(9):e44186. doi: 10.1371/journal.pone.0044186 (PMC3434221; doi:10.1371/journal.pone.0044186)
Supplement: Table S7 — Phylogeny origins of genes involved in phosphorus utilization in biofilm communities. (PDF) [file pone.0044186.s010.pdf]

Table S7. Phylogeny origins of genes involved in phosphorus utilization in biofilm communities

Functional group (genes)	Phylum[a]	Normalized signal intensity[b]			
		24-C	24-S	48-C	48-S
Phytase	*Proteobacteria*	6.7± 1.8	4.7 ± 0.9	4.9 ± 2.0	3.1 ± 0.9
	Ascomycota	8.6 ± 2.0	8.9 ± 0.2	8.4 ± 1.4	6.0 ± 0.8*
	Basidiomycota	1.8 ± 0.2	1.7 ± 0.4	1.8 ± 0.4	1.1 ± 0.8
Exopolyphosphatase (*ppx*)	***Actinobacteria***	20.0 ± 1.9	16.4 ± 2.6*	15.6 ± 2.6	11.8 ± 0.7*
	Bacteroidetes	3.2 ± 2.7	1.1 ± 0.4	1.0 ± 0.3	0.4 ± 0.3*
	Cyanobacteria	17.2 ± 4.2	13.1 ± 2.8	9.4 ± 0.7	7.9 ± 0.4**
	Firmicutes	13.3 ± 0.7	13.1 ± 1.3	12.8 ± 2.0	11.8 ± 1.5
	Proteobacteria	146.0 ± 14.7	137.4 ± 7.2	141.7 ± 28.1	111.4 ± 17.7
	Ascomycota	2.2 ± 0.7	2.4 ± 0.1	3.2 ± 0.7	3.3 ± 0.6

[a]The phyla in bold were those with a significant change in their abundance between the control- and the EcO157-inoculated biofilm at either 24 h or 48 h; [b]The sum of normalized signal intensity for the all DNA probes detected within the same phylum. The t test was performed between the control- and the EcO157-inoculated biofilm (n=3) at each time point and the significance was labeled at 24-S or 48-S (* P<0.1 and ** P<0.05).
